# Optimal allocation to treatments in a sequential multiple assignment randomized trial

**DOI:** 10.1177/09622802211037066

**Published:** 2021-09-23

**Authors:** Andrea Morciano, Mirjam Moerbeek

**Affiliations:** 1 Findomestic Banca, Florence, Italy; 2 Department of Methodology and Statistics, Utrecht University, the Netherlands

**Keywords:** sequential multiple assignment randomized trial trials, optimal allocation, efficiency, response rates, cost constraint, maximin designs

## Abstract

One of the main questions in the design of a trial is how many subjects should be assigned to each treatment condition. Previous research has shown that equal randomization is not necessarily the best choice. We study the optimal allocation for a novel trial design, the sequential multiple assignment randomized trial, where subjects receive a sequence of treatments across various stages. A subject's randomization probabilities to treatments in the next stage depend on whether he or she responded to treatment in the current stage. We consider a prototypical sequential multiple assignment randomized trial design with two stages. Within such a design, many pairwise comparisons of treatment sequences can be made, and a multiple-objective optimal design strategy is proposed to consider all such comparisons simultaneously. The optimal design is sought under either a fixed total sample size or a fixed budget. A Shiny App is made available to find the optimal allocations and to evaluate the efficiency of competing designs. As the optimal design depends on the response rates to first-stage treatments, maximin optimal design methodology is used to find robust optimal designs. The proposed methodology is illustrated using a sequential multiple assignment randomized trial example on weight loss management.

## Introduction

In many randomized controlled trials, participants are equally allocated to intervention arms. Such a design is consistent with the view of clinical equipoise that must exist before the start of the trial.^1^ However, it may be preferable to allocate more participants to one arm than to another, for instance, when variances and/or costs vary across the treatment arms,^1–5^ or when outcomes are categorical rather than quantitative.^6–10^ The derivation of the optimal allocation of units to treatment conditions has not only been done for individually randomized trials, but also for more complex trial designs such as cluster-randomized trials,^11–16^ and trials with partially nested data.^17–19^ From a statistical point of view, it is more efficient to assign more subjects to the condition with the lowest costs and highest variance. Other, more practical, reasons to use unequal allocation over equal allocation include resource constraints, administrative, political or ethical concerns or when the aim is to gain experience from an intervention and to study its feasibility.^5,20^

The focus of these references is on trials where subjects are randomized to either one single treatment or a combination of treatments, but do not change their assigned treatments during the course of the trial. This is a drawback since in real research practice some subjects may benefit more from one treatment and others more from another. Adaptive treatment strategies (ATSs), which are also called dynamic treatment regimens or adaptive interventions, are more flexible in the sense that they allow changing treatments over time.^21–24^ An ATS individualizes treatments to subjects via decision rules that adjust the type, intensity, dosage or delivery of a treatment and specify when, whether and how to proceed at certain critical clinical decisions. For instance, those subjects for whom their assigned treatment turns out to be beneficial may continue the same treatment, while those others may be assigned to another treatment. The use of sequential treatments is often necessary because of: (i) heterogeneous treatment outcomes across subjects, (ii) change in treatment goals over time, (iii) the need to balance potential risks and benefits or (iv) to reduce costs when intensive treatment is not necessary.^25,26^ Also, the use of sequential treatments implies multiple clinical decisions to be taken throughout the course of the study. These clinical decisions are formalized through ATSs.

Based on the number of treatments and treatment switches, various competing ATSs may be developed and they may be compared to one another in a so-called sequential multiple assignment randomized trial (SMART).^25,27^ SMARTs are multi-stage randomized trial designs that are used to inform on the development of multiple ATSs embedded in it. The use of SMART designs allows researchers to evaluate the timing, sequencing and adaptive selection of treatments by using randomization and developing the best sequence(s) of treatments that lead to the optimal outcomes in the long term. In SMARTs, participants are allowed to switch through multiple stages, where each stage corresponds to a clinical decision, and subjects may be randomized at each stage. Sequenced randomization ensures that at each decision point the groups of participants assigned to the intervention options are balanced in terms of patient characteristics. This adds flexibility, allowing participants to remain on those treatments that are having an effect and giving the possibility to switch away to patients being treated with less effective options. This has made SMART designs appealing in a broad variety of health care, behavioural and psychological settings.

Multiple ATSs are embedded in a SMART and the main question in the design phase of a SMART is how many subjects should be assigned to each ATS, and whether an unequal allocation is better than an equal allocation. Some recent papers studied the relation between sample size and power for SMART designs,^25,28–34^ but did not study the optimal allocation of units to treatment sequences and the loss of efficiency of using equal rather than unequal allocation.

The aim of this paper is to derive optimal allocations of units for a prototypical SMART design. This is a two-stage design where all units are randomized to two treatment conditions in the first stage. Those who respond to their assigned treatment are not re-randomized in the second stage, while those who do not respond are re-randomized to two second-stage treatments. This design was considered earlier by NeCamp et al.^32^ in the setting of a cluster-randomized trial. In our contribution, we focus on individual randomization. We focus on sample sizes to be used when comparing two ATSs that start with different first-stage treatments. Four of such pairwise comparisons can be made in their prototypical SMART design, and one comparison may be of more importance than another. We therefore use multiple-objective optimal design methodology to consider all comparisons simultaneously, while taking into account their relative importance.^35^ Multiple-objective optimal designs are useful when the study has multiple and conflicting objectives, such multiple pairwise comparisons of marginal means of ATSs in a SMART. It combines these objectives in one optimality criterion and tries to seek a design that is highly efficient for each of these criteria. We provide a Shiny App to calculate the optimal allocation of units and to evaluate the efficiency of the design with equal allocation. We demonstrate our optimal design methodology on the basis of a SMART example that compares two different treatments, nutrition (NUT) and physical activity (PHY), for weight loss management. Our focus is on SMARTs with a quantitative outcome with individual randomization. In other words, we do not focus on cluster-randomized SMARTs or other complex SMART designs with clustered data.

The remainder of our contribution is organized as follows. Section ‘Prototypical SMART design’ further discusses the prototypical SMART design and its embedded ATSs. Furthermore, this section introduces the example of weight loss management. Section ‘Derivation of the optimal design’ derives the optimal allocation of units for studies in which either the total sample size or the budget is fixed. In the latter case, we consider the realistic situation where costs may vary across treatment conditions. The optimal allocation turns out to depend on the subjects’ probabilities to respond to their first-stage treatment. We therefore also focus on maximin optimal designs that are robust to incorrect prior estimates of these probabilities. Furthermore, Section ‘Derivation of the optimal design’ introduces the Shiny App that we developed for finding the optimal design. Section ‘A SMART example’ demonstrates our optimal design methodology on the basis of the weight loss example. It shows how the optimal design is influenced by the costs per treatment, proportion of responders to first-stage treatments and the relative importance of the four pairwise comparisons. Section ‘Discussion’ summarizes our findings, discusses limitations of this contribution and gives directions for future research.

## Prototypical SMART design

Before we focus on the prototypical SMART, we rehearse some general ingredients for arbitrary SMART (see for instance Ertefaie et al.,^36^ but using different notation). The observed covariates and treatment assignment at stage *k* are denoted $O_{k}$ and $X_{k}$ , respectively, and ${\bar{O}}_{k}$ and ${\bar{X}}_{k}$ denote the covariate and treatment histories up to and including stage *k*. Within a SMART multiple ATSs are embedded; these are denoted $d_{i}$ , i=1,…,I . An ATS is basically a treatment trajectory and denoted by a vector of counterfactual treatment assignments for a given individual *j*. If the SMART has two stages, then $d_{i}=(X_{1},X_{2}^{R},X_{2}^{\mathrm{NR}})$ , where $X_{2}^{R}$ is the treatment assignment in the second stage had the subject responded, and $X_{2}^{\mathrm{NR}}$ is the treatment assignment in the second stage had he or she not responded. So, for a subject who responds, $X_{2}^{\mathrm{NR}}$ is not observed, and for a subject who does not respond $X_{2}^{R}$ , hence $d_{i}$ is called a vector of counterfactual treatments. The observed treatment history only includes the treatments a subject has actually been assigned to ${\bar{X}}_{2}=(X_{1},X_{2})$ . At the end of each stage *k*, a tailoring variable is measured which determines if a subject has responded to the treatment in that stage or not. In other words, this variable determines which treatment the subject is assigned to in the subsequent stage. At the end of the study (i.e. at the end of the final stage) the continuous outcome variable $Y_{j}$ is measured on each subject. These outcomes are then used to compare different ATSs to one another.

**Figure 1.**
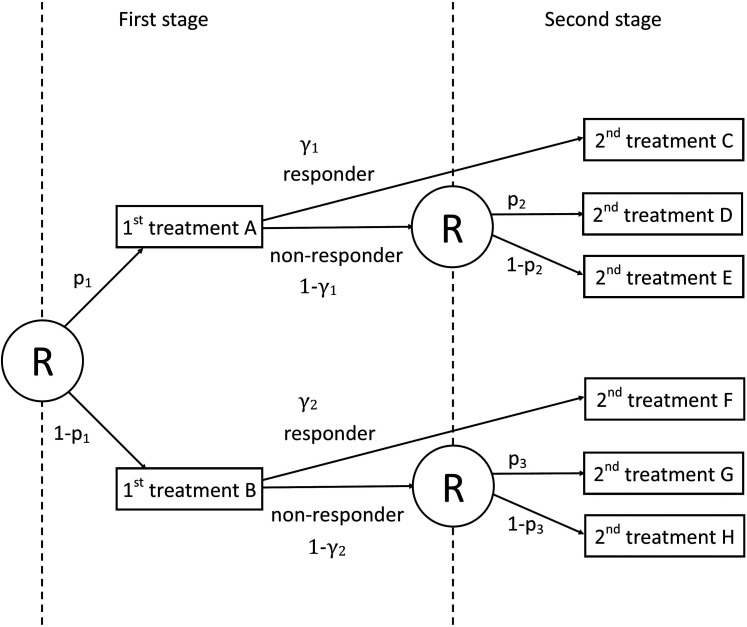
A scheme of the prototypical sequential multiple assignment randomized trial (SMART) design from NeCamp et al.^32^ Circled ‘R’ denotes randomization at each stage. *p*_1_ and (1 − *p*_1_) are, respectively, the proportions of subjects receiving first-stage treatments A and B. *p*_2_ and (1 − *p*_2_) are, respectively, the proportions of subjects receiving second-stage treatments D and E for non-responders starting with first-stage treatment A. *p*_3_ and (1 − *p*_3_) are, respectively, the proportions of subjects receiving second-stage treatments G and H for non-responders starting with first-stage treatment B. *γ*_1_ and *γ*_2_ indicate, respectively, response rates for the first-stage treatments A and B.

The prototypical SMART design is visualized in Figure 1. This design has been used in various research fields; published examples of its use in the treatment and long-term management of many chronic conditions include weight loss,^26,37,38^ substance abuse,^39,40^ cancer research,^41,42^ adolescent depression,^43^ adolescent conduct problems,^44^ suicide,^45^ and attention-deficit/hyperactivity disorder.^46^

The prototypical SMART is a two-stage design with two first-stage treatments A and B; the proportions randomized to these treatments are denoted $p_{1}$ and $1-p_{1}$ , respectively. After some amount of time it is determined which subjects respond to their first-stage treatment, depending on some criterion such as a sufficient amount of weight loss or smoking cessation. The response rates to first-stage treatments A and B are equal to $\gamma_{1}$ and $\gamma_{2}$ , respectively. Those subjects who respond to their first-stage treatment are not further randomized, but receive second-stage treatment C or F, depending on their first-stage treatment. This may be the same as the first-stage treatment, but may also be another treatment or discontinuation of treatment with or without further monitoring. Those subjects who do not respond to their first-stage treatment are further randomized. Non-responders who received first-stage treatment A are randomized to second-stage treatments D and E, with proportions $p_{2}$ and $1-p_{2}$ , respectively. Such a second-stage treatment may be an intensified version of the first-stage treatment A, treatment A augmented with another treatment (which may be first-stage treatment B), first-stage treatment B, or an entirely different treatment. In the same manner, non-responders who received first-stage treatment B are randomized to two second-stage treatments G and H. This design includes eight different treatment conditions, where some of the second-stage treatments may be the same as the first-stage treatments or a combination of them.

Four ATSs are embedded in the prototypical SMART design, see Table 1. For instance, the first ATS, denoted $d_{1}$ , assigns all subjects to first-stage treatment A. Responders receive second-stage treatment C while non-responders receive second-stage treatment D.

**Table 1.** The four ATSs embedded in the prototypical SMART design.

ATS label	First-stage treatment	Status at the end of first-stage	Second-stage treatment
$d_{1}$	A	Responder	C
		Non-responder	D
$d_{2}$	A	Responder	C
		Non-responder	E
$d_{3}$	B	Responder	F
		Non-responder	G
$d_{4}$	B	Responder	F
		Non-responder	H

ATS: adaptive treatment strategy; SMART: sequential multiple assignment randomized trial.

The primary analysis goal of a SMART design is usually one of the following: (i) comparing first-stage intervention options; (ii) comparing second-stage intervention options; (iii) comparing two or more embedded ATSs in the study starting with the same first-stage intervention option or (iv) comparing two or more embedded ATSs in the study starting with different first-stage intervention options.^31^ In the derivation of our optimal design, we focus on embedded ATSs that start with different first-stage treatments, which is a common primary aim in SMARTs.^32^

### Example: weight loss management

Bariatric surgery is an effective treatment for obese patients to lose weight. Given its costs, potentially harmful side effects and the risk of death, patients in the Netherlands are only considered eligible if they can demonstrate they have previously attempted other means to lose weight. Two treatments are an increase in PHY and a change in NUT.

**Figure 2.**
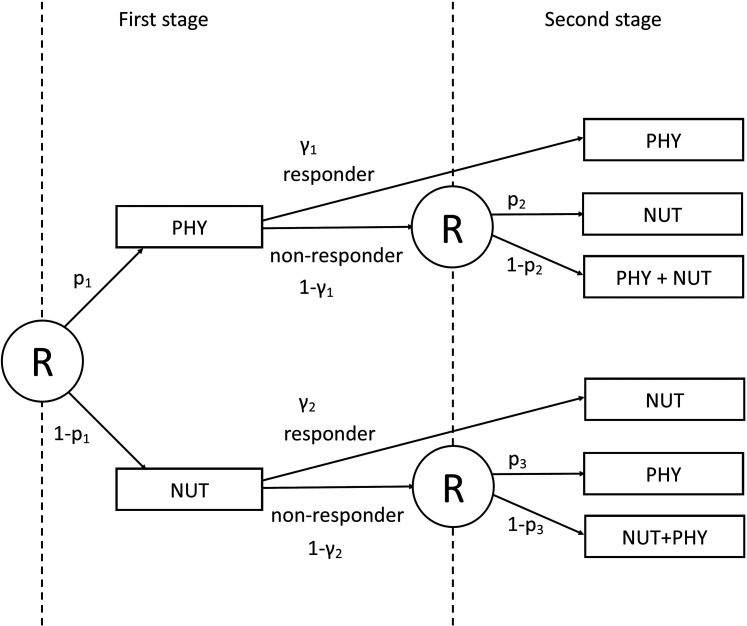
A scheme of the example SMART design on weight loss. Circled ‘R’ denotes randomization at each stage. *p*_1_ and (1 − *p*_1_) are, respectively, the proportions of subjects receiving the two first-stage treatments: PHY and NUT. *p*_2_ and (1 − *p*_2_) are, respectively, the proportions of subjects receiving second-stage treatments NUT and NUT + PHY for subjects starting with PHY as first-stage treatment. *p*_3_ and (1 − *p*_3_) are, respectively, the proportions of subjects receiving second-stage treatments PHY and NUT + PHY for subjects starting with NUT as first-stage treatment. *γ*_1_ and *γ*_2_ indicate, respectively, response rates for the first-stage treatments PHY and NUT.

SMART: sequential multiple assignment randomized trial; PHY: physical activity; NUT: nutrition.

Figure 2 visualises the example SMART design. All patients are first randomized to either PHY or NUT. Then, at the end of the first stage, subjects are categorized as responders or non-responders, according to some predefined definition of response, for example, a threshold for weight loss after a given period of time. Non-responders are then re-randomized to second-stage treatments, regardless of their treatment in the first-stage. They either switch to the other treatment or pursue with a combination of both treatments (NUT + PHY) in the second stage. Responders are not re-randomized and pursue with their first-stage treatment. This example is visualized in Figure 2. Four different ATSs are embedded within this prototypical SMART design: (i) $d_{1}=(\mathrm{PHY},\;\mathrm{PH}Y^{R},\mathrm{NU}T^{\mathrm{NR}})$ , (ii) $d_{2}=(\mathrm{PHY},\;\mathrm{PH}Y^{R},{\left(\mathrm{NUT}+\mathrm{PHY}\right)}^{\mathrm{NR}})$ , (iii) $d_{3}=(\mathrm{NUT},\;\mathrm{NU}T^{R},\mathrm{PH}Y^{\mathrm{NR}})$ and (iv) $d_{4}=(\mathrm{NUT},\;\mathrm{NU}T^{R},{\left(\mathrm{NUT}+\mathrm{PHY}\right)}^{\mathrm{NR}})$ . The superscript ^R^ refers to second-stage treatment assigned to responders, while the superscript ^NR^ denotes second-stage treatment assigned to non-responders.

The SMART design of this example is a simplification of the prototypical SMART design in the sense that just two treatments are involved. Responders continue with their first-stage treatment, while non-responders are randomized to the other treatment or a combination of both treatments. This specific SMART design was previously used for, among others, the treatment of anxiety disorder,^25^ obsessive–compulsive disorder^47^ and chronic pain.^48^

## Derivation of the optimal design

### Introduction

For a given ATS $d_{i},\;i=1,\;\ldots ,\;4$ , let $Y_{j},\;j=1,\;\ldots ,\;N_{d_{i}}$ be the continuous primary outcome of interest for the *j*th subject as measured at the end of stage 2, with $N_{d_{i}}$ denoting the number of subjects whose treatment trajectories are consistent with the ATS $d_{i}$ . $Y_{j}$ is supposed to have $E(Y_{j})=\mu_{i}$ and $Var(Y_{j})=\;\sigma^{2}$ , for all $j=1,\;\ldots ,\;N_{d_{i}}$ . We assume common variance $\;\sigma^{2}$ across all four ATSs. The target parameter $\mu_{i}$ , the marginal mean outcome expected under ATS $d_{i}$ , depends on the proportion of responders to first-stage treatment in ATS $d_{i}$ in the population. It is estimated by a weighted average of the observed outcomes of subjects whose treatment trajectories are consistent with $d_{i}$ .^31^

The weights follow from the fact that there is a structural imbalance between responders and non-responders: the non-responders are re-randomized but the responders are not. For instance, for ATS $d_{1}$ , responders have a probability of $p_{1}$ of receiving the treatment sequence they actually received, and their subject-specific weights are $W_{j}=1/p_{1}$ . For non-responders, this probability is $p_{1}p_{2}$ and hence their weight is $W_{j}=1/p_{1}p_{2}$ . Here $p_{1}$ is the randomization probability to treatment A in the first-stage and $p_{2}$ is the randomization probability to treatment C in the second-stage. The weights are the inverse of the probabilities, hence the weighting is called inverse probability weighting. By using these weights, the relative contribution of the responders and non-responders in the calculation of the weighted mean outcome in ATS $d_{1}$ is the same as when this ATS had not been embedded in a SMART. In other words, since the ATS is embedded in a SMART, the non-responders have a higher weight than the responders to account for the fact that some of them are randomized to treatment E, rather than treatment D. This is a generalization from the work of Ghosh et al.^31^ in the sense that we allow the proportions $p_{1}$ and $p_{2}$ to be unequal to 0.5. For the other ATSs, subject-specific weights can be obtained in a similar way.

The weighted mean for the continuous primary outcome of interest for ATS $d_{i}$ is equal to

(1) $$\begin{array}{c} {\bar{Y}}_{d_{i}}=\frac{\sum_{j}^{N_{d_{i}}}\;W_{j}Y_{j}\;}{\sum_{j}^{N_{d_{i}}}\;W_{j}\;} \end{array}$$

The expected value of this weighted mean is given by

(2) $$\begin{array}{c} E({\bar{Y}}_{d_{i}})=\frac{\sum_{j}^{N_{d_{i}}}\;W_{j}E(Y_{j})\;}{\sum_{j}^{N_{d_{i}}}\;W_{j}\;}=\mu_{i} \end{array}$$

Equation (2) shows that the weighted mean is an unbiased estimator of the marginal mean. The variance of the weighted mean is equal to

(3) $$\begin{array}{c} \mathrm{Var}({\bar{Y}}_{d_{i}})=\;\sigma^{2}\frac{\sum_{j}^{N_{d_{i}}}\;W_{j}^{2}\;}{{\left(\sum_{j}^{N_{d_{i}}}\;W_{j}\;\right)}^{2}} \end{array}$$

For each ATS $d_{i}$ , the variance of the weighted mean is computed using the subject-specific weights. First, the expected number of people in the trial whose treatment trajectories are consistent with $d_{i}$ is computed for each ATS. For $d_{1}$ , this is equal to

(4) $$\begin{array}{c} E(N_{d_{1}})=Np_{1}\gamma_{1}+Np_{1}(1-\gamma_{1})p_{2}, \end{array}$$

with the first term on the right side representing the expected number of responders and the second being the expected number of non-responders. The proportions $p_{1}$ and $p_{2}$ are defined as above, while *N* is the total sample size of the SMART and $\gamma_{1}$ is the response rate to first-stage treatment A.

Following from (4), we obtain

(5) $$\begin{array}{c} \sum_{j}^{N_{d_{1}}}\;W_{j}=\;\frac{1}{\;p_{1}}Np_{1}\gamma_{1}+\frac{1}{\;p_{1}p_{2}}Np_{1}(1-\gamma_{1})p_{2}=N \end{array}$$

(6) $$\begin{array}{c} \sum_{j}^{N_{d_{1}}}\;W_{j}^{2}=\frac{1}{\;p_{1}^{2}}Np_{1}\gamma_{1}+\frac{1}{{\left(\;p_{1}p_{2}\right)}^{2}}Np_{1}(1-\gamma_{1})p_{2}=\frac{N\gamma_{1}}{\;p_{1}}+\frac{N(1-\gamma_{1})}{\;p_{1}p_{2}} \end{array}$$

The variance for the weighted mean ${\bar{Y}}_{d_{1}}$ , for ATS $d_{1}$ , is obtained by plugging (5) and (6) into (3):

(7) $$\begin{array}{c} \mathrm{Var}({\bar{Y}}_{d_{1}})=\frac{\sigma^{2}}{N}\frac{\gamma_{1}p_{2}+(1-\gamma_{1})}{\;p_{1}p_{2}} \end{array}$$

The right side of (7) consists of two factors. The first is the common variance of a mean, while the second is used to account for the fact that subjects may be re-randomized. This second factor is a function of the response rate $\gamma_{1}$ to first-stage treatment A.

Using their respective subject-specific weights, formulae for the variance of the weighted mean ${\bar{Y}}_{d_{i}}$ for the other ATSs are obtained in a similar way; these are shown in Table 2.

**Table 2.** Variance for the weighted mean $\;{\bar{Y}}_{d_{i}}$ for the four adaptive treatment strategies (ATSs) embedded.

$d_{i}$	$\mathrm{Var}({\bar{Y}}_{d_{i}})$
$d_{1}=(A,C^{R},D^{\mathrm{NR}})$	$\sigma^{2}\frac{\gamma_{1}p_{2}+(1-\gamma_{1})}{Np_{1}p_{2}}$
$d_{2}=(A,\;C^{R},E^{\mathrm{NR}})$	$\sigma^{2}\frac{\gamma_{1}(1-p_{2})+(1-\gamma_{1})}{Np_{1}(1-p_{2})}$
$d_{3}=(B,\;F^{R},G^{\mathrm{NR}})$	$\sigma^{2}\frac{\gamma_{2}p_{3}+(1-\gamma_{2})}{N(1-p_{1})p_{3}}$
$d_{4}=(B,\;F^{R},H^{\mathrm{NR}})$	$\sigma^{2}\frac{\gamma_{2}(1-p_{3})+(1-\gamma_{2})}{N(1-p_{1})(1-p_{3})}$

We consider pairwise comparisons of ATSs that start with different first-stage treatments. The expected difference in weighted means of two such ATSs $d_{i}$ and $d_{i^{\prime }}$ (with i=1or2 and $i^{\prime }=\;3\;\mathrm{or}\;4$ ) is $\mu_{i}-\mu_{i^{\prime }}$ with the corresponding variance

(8) $$\begin{array}{c} \mathrm{Var}({\bar{Y}}_{d_{i}}-{\bar{Y}}_{d_{i^{\prime }}})=\mathrm{Var}({\bar{Y}}_{d_{i}})+\mathrm{Var}({\bar{Y}}_{d_{i^{\prime }}})+2\mathrm{Cov}({\bar{Y}}_{d_{i}},{\bar{Y}}_{d_{i^{\prime }}})=\mathrm{Var}\;({\bar{Y}}_{d_{i}})+\mathrm{Var}({\bar{Y}}_{d_{i^{\prime }}}) \end{array}$$

where $\mathrm{Cov}({\bar{Y}}_{d_{i}},{\bar{Y}}_{d_{i^{\prime }}})=0$ if we assume that weighted means of ATSs that start with different first-stage treatments are independent. This assumption holds as long as outcomes of subjects from ATSs that start different first-stage treatments are independent

Considering the ATSs embedded in our example, four possible pairwise comparisons exist, with corresponding variances: (i) $\Phi_{13}=\mathrm{Var}({\bar{Y}}_{d_{1}}-{\bar{Y}}_{d_{3}})$ ; (ii) $\Phi_{14}=\mathrm{Var}({\bar{Y}}_{d_{1}}-{\bar{Y}}_{d_{4}})$ ; (iii) $\Phi_{23}=\mathrm{Var}({\bar{Y}}_{d_{2}}-{\bar{Y}}_{d_{3}})$ and (iv) $\Phi_{24}=\mathrm{Var}({\bar{Y}}_{d_{2}}-{\bar{Y}}_{d_{4}})$ , with ${\bar{Y}}_{d_{i}}$ being the weighted mean for the continuous primary outcome variable of interest for the ATS $d_{i},\;i=1,\ldots ,4$ . Formulae for the variance of these comparisons can be derived by plugging in the variances of the single ATSs as reported in Table 2.

The optimal design $\xi_{ii^{\prime }}^{*}=(\;p_{1}^{*},p_{2}^{*},p_{3}^{*})$ for objective $\Phi_{ii^{\prime }}$ is the one among all designs $\xi =(\;p_{1},p_{2},p_{3})$ in the design space $\Omega =(0\leq p_{1}\leq 1,0\leq p_{2}\leq 1,0\leq p_{3}\leq 1)$ for which $\mathrm{Var}({\bar{Y}}_{d_{i}}-{\bar{Y}}_{d_{i^{\prime }}})$ is minimized. Each objective has its own optimal design. For instance, the optimal design for $\Phi_{13}$ is $\xi_{13}^{*}=(0.5,1,1)$ , which implies both first-stage treatments have randomization probability 0.5, all non-responders in first-stage treatment A receive second-stage treatment D, and all non-responders to first-stage treatment B receive second-stage treatment G. The optimal designs for the other objectives are $\xi_{14}^{*}=(0.5,1,0)$ , $\xi_{23}^{*}=(0.5,0,1)$ and $\xi_{24}^{*}=(0.5,0,0)$ . The optimal design for one objective does not only hold for the other single objectives, but it may also perform poorly.^49^ For that reason, a multiple-objective optimal design is used, so that all of the four pairwise comparisons are taken into account simultaneously. We do so by using a weighted sum of the four objectives, where weights are to be chosen by the user. The use of weights allows placing more emphasis on the one objective than another, subject to the researcher's interests and the goals of the study. A constraint is put on the weights such that their sum is equal to 1. The optimal design problem becomes a multiple-objective optimal design problem. The aim is to minimize the optimality criterion

(9) $$\begin{array}{c} \begin{array}{c} \begin{array}{c} \Phi =\;\Phi_{13}\lambda_{13}+\Phi_{14}\lambda_{14}+\;\Phi_{23}\lambda_{23}+\Phi_{24}\lambda_{24} \end{array} \end{array} \end{array}$$

with $\lambda_{ii^{\prime }}$ being the weight assigned to the respective objective $\Phi_{ii^{\prime }}$ . The corresponding optimal design is a so-called compound-optimal design.

### Optimal design under a fixed total sample size

In this scenario, the optimal design is sought under a fixed total sample size *N*. This is a realistic scenario when studying treatments for a rare disease or condition, but it can also be used when resource constraints allow recruiting a fixed number of subjects. It is assumed that a priori estimates of the response rates $\gamma_{1}$ and $\gamma_{2}$ are available.

The optimal design minimizes the objective in (9); it is found by taking the gradient of (9) with respect to $p_{1}$ , $p_{2}$ and $p_{3}$ . The optimal proportions for the second-stage treatments are given by

(10) $$\begin{array}{c} \;p_{2}^{*}=\frac{\sqrt{\left(\lambda_{13}+\lambda_{14}\right)}}{\sqrt{\left(\lambda_{23}+\lambda_{24}\right)}+\sqrt{\left(\lambda_{13}+\lambda_{14}\right)}} \end{array}$$

and

(11) $$\begin{array}{c} \;p_{3}^{*}=\frac{\sqrt{\left(\lambda_{13}+\lambda_{23}\right)}}{\sqrt{\left(\lambda_{13}+\lambda_{23}\right)}+\sqrt{\left(\lambda_{14}+\lambda_{24}\right)}} \end{array}$$

It is worth noting that the optimal second-stage proportions $p_{2}^{*}$ and $p_{3}^{*}$ do not depend on the response rates $\gamma_{1}$ and $\gamma_{2}$ , or on the total sample size *N*, but only on the choice of the weights. In particular, $p_{2}^{*}$ increases as $\lambda_{13}$ and/or $\lambda_{14}$ increase. This is obvious since objectives $\Phi_{13}$ and $\Phi_{14}$ are comparisons that include treatment D, and more efficient comparisons can be made if more subjects are assigned to this treatment. Similarly, $p_{3}^{*}$ increases when $\lambda_{13}$ and/or $\lambda_{23}$ increase. This is also obvious since objectives $\Phi_{13}$ and $\Phi_{23}$ are comparisons that include treatment G, and more efficient comparisons can be made if more subjects are assigned to this treatment.

The optimal randomization probability for the first-stage treatment A takes on a more complicated form:

(12) $$\begin{array}{c} \;p_{1}^{*}=\frac{\sqrt{\varphi }}{\sqrt{\psi }+\sqrt{\varphi }} \end{array}$$

where

(13) $$\begin{array}{c} \psi =\;[(\gamma_{2}p_{3}^{*}+(1-\gamma_{2}))(\lambda_{13}+\lambda_{23})(1-p_{3}^{*})+(\gamma_{2}(1-p_{3}^{*})+(1-\gamma_{2}))(\lambda_{14}+\lambda_{24})p_{3}^{*}]p_{2}^{*}(1-p_{2}^{*})\; \end{array}$$

(14) $$\begin{array}{c} \varphi =\;[(\gamma_{1}p_{2}^{*}+(1-\gamma_{1}))(\lambda_{13}+\lambda_{14})(1-p_{2}^{*})+(\gamma_{1}(1-p_{2}^{*})+(1-\gamma_{1}))(\lambda_{23}+\lambda_{24})p_{2}^{*}]p_{3}^{*}(1-p_{3}^{*}) \end{array}$$

$p_{1}^{*}$ depends on both $\gamma_{1}$ and $\gamma_{2}$ , and on the optimal proportions $p_{2}^{*}$ and $p_{3}^{*}$ , while it does not depend on *N*. A detailed derivation of the optimal design is given in the online supplement.

### Optimal design under a fixed budget

In this scenario, we consider a budgetary constraint: the total costs *C* for treating subjects should not exceed the budget *B*. The costs are calculated as

(15) $$\begin{array}{c} C=\;c_{A}N_{A}+c_{B}N_{B}+\cdots +c_{H}N_{H} \end{array}$$

where $c_{A}$ are the costs per subject in treatment A and $N_{A}$ are the number of subjects who receive treatment A, and similarly for the other treatments B to H. The costs may vary across subjects and are assumed to be known beforehand. The sample sizes are stochastic since they depend on the proportions $p_{1}$ , $p_{2}$ and $p_{3}$ and response rates $\gamma_{1}$ and $\gamma_{2}$ . In the derivation of the optimal design, we use their expected values. For the first-stage treatments, we have

(16) $$\begin{array}{c} E(N_{A})=p_{1}N\;\mathrm{and}\;E(N_{B})=(1-p_{1})N \end{array}$$

for the second-stage treatments C, D and E, we have

(17) $$\begin{array}{c} E(N_{C})=\gamma_{1}p_{1}N\;\;\mathrm{and}\;\;E(N_{D})=\gamma_{1}p_{1}p_{2}N\;\;\mathrm{and}\;\;E(N_{E})=\gamma_{1}p_{1}(1-p_{2})N \end{array}$$

and for the second-stage treatments F, G and H, we have

(18) $$\begin{array}{c} E(N_{F})=\gamma_{2}(1-p_{1})N\;\;\mathrm{and}\;\;E(N_{G})=\gamma_{2}(1-p_{1})p_{3}N\;\;\mathrm{and}\;\;E(N_{H})=\gamma_{2}(1-p_{1})(1-p_{3})N \end{array}$$

For a given budget, the total sample size *N* that can be used decreases when the costs increase. This implies that a design is not only determined by the proportions but also by the total sample size: $\xi =(\;p_{1},p_{2},p_{3},N)$ . The optimal design is found in a numerical manner through a domain search algorithm, see the online supplement for more details.

### Robust optimal design

The optimal design depends on the response rates $\gamma_{1}$ and $\gamma_{2}$ , hence the optimal design is locally optimal. These parameters are often unknown in the design stage of a SMART and an educated a priori guess based on expert opinions or findings in the literature should be used. There is, however, no guarantee that such a guess is correct and robust optimal design methodology may be used to protect against a loss of efficiency due to a misspecification of the response rates. We use maximin optimal design methodology^50^ to allow specification of intervals, rather than point estimates, of the two response rates.

The maximin optimal design $\xi_{\mathrm{MMD}}$ maximizes the minimal relative efficiency (RE) among all designs in the design space Ω . In other words, it selects the best of the worst-case scenarios. The maximin optimal design can be found using the following three steps:

1. Define the parameter space for the response rates and the design space Ω for the proportions. For instance, the first response rate $\gamma_{1}$ may be between 0.2 and 0.3 and the second response rate $\gamma_{2}$ may be between 0.35 and 0.45. The design space is $\Omega =(0\leq p_{1}\leq 1,\;0\leq p_{2}\leq 1,\;0\leq p_{3}\leq 1)$ .
2. For each possible combination of the two response rates in the parameter space, compute the locally optimal design $\xi_{\mathrm{LOD}}$ . Then compute the RE of each design ξ in Ω compared with the locally optimal design: $\mathrm{RE}=\Phi (\xi_{\mathrm{LOD}})/\Phi (\xi )$ .
3. For each design in Ω , find its smallest RE value within the parameter space. Then, select the design that has the highest minimum RE across all designs in the design space. This is the maximin optimal design $\xi_{\mathrm{MMD}}$ and its minimum RE is called the maximin value.

This procedure yields the design which is most robust to a misspecification of the response rates and it can be used when working under a fixed budget or under a fixed total sample size.

### Statistical power for the optimal design

Once the optimal allocation to treatments has been derived, it makes sense to determine how much power the study has for each of the four pairwise comparisons of ATSs ^51^. The following steps should be taken in such a power analysis:

1. Calculate the variance $\mathrm{Var}({\bar{Y}}_{d_{i}})$ for each of the four ATSs in the SMART. For the case of a fixed total sample size this can be done easily by plugging in the optimal proportions $p_{1}^{*}$ , $p_{2}^{*}$ and $p_{3}^{*}$ and total sample size *N* into the equations of Table 2. For the case of a fixed budget, first, the total sample size *N* has to be calculated from the budget, costs and optimal proportions. This can be done on the basis of equations (15) to (18), as is further explained in the online supplement.
2. For each of the four pairwise comparisons of ATSs: calculate $\mathrm{Var}({\bar{Y}}_{d_{i}}-{\bar{Y}}_{d_{i^{\prime }}})$ from equation (8).
3. For each of the four pairwise comparisons of ATSs, get a prior estimate of the expected difference in marginal means $\mu_{i}-\mu_{i^{\prime }}$ . A prior estimate may be obtained from the literature or an expert's expectations. As an alternative, one may use the minimal relevant effect size, that is, the smallest effect size that is considered to be relevant.
4. For each of the four pairwise comparisons of ATSs, select the type I error rate α and decide whether a one-sided or two-sided test has to be performed.
5. For each of the four pairwise comparisons of ATSs, calculate the power. For a one-sided alternative use the following equation:
$$1-\beta =z^{-1}\left(\frac{\left|\mu_{i}-\mu_{i^{\prime }}\right|}{\mathrm{Var}({\bar{Y}}_{d_{i}}-{\bar{Y}}_{d_{i^{\prime }}})}-z_{1-\alpha }\right)$$

where $\mathrm{Var}({\bar{Y}}_{d_{i}})$ and $\mathrm{Var}({\bar{Y}}_{d_{i^{\prime }}})$ are the variances of the two ATSs to be compared, $z_{1-\alpha }$ is the (1−α)th quantile of the standard normal distribution and $z^{-1}$ is the inverse of the standard normal distribution. For a two-sided alternative, α has to be replaced by α/2 .

### Shiny app

We developed a Shiny app^52^ to facilitate finding the optimal design; it is available from https://andreamorciano.shinyapps.io/OptimalSMART/. It calculates locally optimal designs for a fixed total sample size as well as a fixed budget. In the first case, the user should specify the total sample size, in the latter case, he or she should specify the costs per treatment along with the budget. Furthermore, an a priori estimate of the two response rates should be specified to find the locally optimal design. The numerical algorithm that finds the optimal design for the budgetary constraint has a precision of 0.00002 for the optimal proportions.

The Shiny app can also be used to find the maximin optimal design. It that case intervals $\left[\gamma_{1}-0.05,\gamma_{1}+0.05\right]$ and $\left[\gamma_{2}-0.05,\gamma_{2}+0.05\right]$ are considered around the user-specified values $\gamma_{1}$ and $\gamma_{2}$ . These intervals are continuous; in our algorithm, we use a step size of 0.01 to discretize these intervals, while a step size of 0.05 is used for the response rates. In the case the reader is interested in using a different step size, he/she can contact the first author.

## A SMART example

### Introduction

We apply the optimal design methodology to the example of the weight loss management study of Figure 2. Participants are randomized to two first-stage treatments: PHY and NUT. A response is defined as a (absolute or relative) loss in body weight that exceeds a user-selected threshold value. We use three sets of a priori guesses for the two response rates of the two first-stage treatments: $\left(\gamma_{1},\gamma_{2})=(0.15,0.25\right)$ , $\left(\gamma_{1},\gamma_{2})=(0.25,0.40\right)$ and $\left(\gamma_{1},\gamma_{2})=(0.40,0.55\right)$ . In each case, we choose a larger value for NUT than for PHY, as previous research has demonstrated that PHY produces smaller bodyweight loss than diet (NUT).^53^ For the first set of response rates, the definition of a response is most stringent, resulting in the smallest response rates, and for the third it is most lenient, resulting in the highest response rates.

We consider three sets of weights for the multiple-objective optimal design (9). The first considers each comparison to be of equal importance, which implies that equal weights are used: $\left(\lambda_{13},\lambda_{14},\lambda_{23},\lambda_{24})=(0.25,\;0.25,\;0.25,\;0.25\right)$ . The second puts more emphasis on those comparisons where second-stage treatments are either PHY or NUT, but not a combination of the two. In this case, researchers are mainly interested in the comparison between $d_{1}=(\mathrm{PHY},\;\mathrm{PH}Y^{R},\mathrm{NU}T^{\mathrm{NR}})$ and $d_{3}=(\mathrm{NUT},\;\mathrm{NU}T^{R},\mathrm{PH}Y^{\mathrm{NR}})$ rather than the other ones. Designs with a single second-stage treatment are less expensive, they may be easier to implement by the researchers and easier to adhere to by the participants. As an illustration we use $\left(\lambda_{13},\lambda_{14},\lambda_{23},\lambda_{24})=(0.70,\;0.10,\;0.10,\;0.10\right)$ . The third set of weights puts more emphasis on those second-stage treatments that are a combination of NUT and PHY, for instance, because there is a believe combined treatment is more effective. In that case the main focus is on the comparison between $d_{2}=(\mathrm{PHY},\;\mathrm{PH}Y^{R},{\left(\mathrm{NUT}+\mathrm{PHY}\right)}^{\mathrm{NR}})$ and $d_{4}=(\mathrm{NUT},\;\mathrm{NU}T^{R},{\left(\mathrm{NUT}+\mathrm{PHY}\right)}^{\mathrm{NR}})$ . As an illustration we use $\left(\lambda_{13},\lambda_{14},\lambda_{23},\lambda_{24})=(0.10,\;0.10,\;0.10,\;0.70\right)$ .

For this specific example, we developed another version of our Shiny app; this is available at https://andreamorciano.shinyapps.io/OptimalSMART2/.

### Locally optimal design under a fixed total sample size

For each combination of $(\gamma_{1},\gamma_{2})\;$ and $\left(\lambda_{13},\lambda_{14},\lambda_{23},\lambda_{24}\right)$ , the optimal design is given in Table 3, along with the RE of the balanced design (where $p_{1}=p_{2}=p_{3}=0.50$ ) as compared to the optimal design. We observe the optimal design hardly depends on the response rates, but it does depend on the weights. For each set of weights, the optimal design dictates (about) equal randomization to first-stage treatments. For the first set of weights, the optimal design is (almost) equal to the balanced design and the RE of the balanced design is 1. For the second set of weights more than half (two-thirds) of participants are randomized to single second-stage treatments. This is obvious because the chosen weights put more emphasis on the comparison of single second-stage treatments. For the third set of weights, less than half (one-third) of participants are randomized to single second-stage treatments. This is also obvious because the chosen weights put more emphasis on the comparison of combined second-stage treatments. The optimal proportions $p_{2}^{*}$ and $p_{3}^{*}$ for the second set of weights are the complement of those for the third set of weights. In all cases, the RE of the balanced design is above 0.9, which implies it performs rather well as compared to the optimal design.

**Table 3.** Locally optimal design: optimal proportions for first-stage $(p_{1}^{*})\;$ and second-stage $(p_{2}^{*},\;p_{3}^{*})\;$ treatments for three different sets of weights $\left(\lambda_{13},\lambda_{23},\lambda_{14},\lambda_{24}\right)$ for the multiple-objective optimal design, and for three different sets of response rates $\left(\gamma_{1},\gamma_{2}\right)$ . The relative efficiency (RE) of the balanced design is also provided. The optimal proportions are derived under a fixed total sample size.

		$(\lambda_{13},\lambda_{23},\lambda_{14},\lambda_{24})=$ (0.25,0.25,0.25,0.25)				$(\lambda_{13},\lambda_{23},\lambda_{14},\lambda_{24})=$ (0.70,0.10,0.10,0.10)				$(\lambda_{13},\lambda_{23},\lambda_{14},\lambda_{24})=$ (0.10,0.10,0.10,0.70)			
$\gamma_{1}$	$\gamma_{2}$	$p_{1}^{*}$	$p_{2}^{*}$	$p_{3}^{*}$	RE	$p_{1}^{*}$	$p_{2}^{*}$	$p_{3}^{*}$	RE	$p_{1}^{*}$	$p_{2}^{*}$	$p_{3}^{*}$	RE
0.15	0.25	0.50	0.50	0.50	1	0.50	0.67	0.67	0.91	0.50	0.33	0.33	0.91
0.25	0.40	0.51	0.50	0.50	1	0.51	0.67	0.67	0.92	0.51	0.33	0.33	0.92
0.40	0.55	0.51	0.50	0.50	1	0.51	0.67	0.67	0.93	0.51	0.33	0.33	0.93

The results do not necessarily apply to other combinations of weights and response rates, so a researcher who is planning a SMART is advised to use our Shiny app to derive the optimal design for the trial at hand, and to do a sensitivity analysis to study how the optimal design is influenced using by various realistic combinations of weights and response rates.

### Locally optimal design under a fixed budget

To find the optimal design under a budgetary constraint, the costs for both treatments and the budget need to be defined. We assume both stages are of equal length, so the costs do not vary across stages. The costs for combined treatment are the sum of the costs for both single treatments. We consider two sets of costs for NUT ( $C_{N}$ ) and PHY ( $C_{P}$ ): $\left(C_{N},C_{P})=(300,50\right)$ and $\left(C_{N},C_{P})=(300,300\right)$ . Let us assume the costs are expressed in euros and the length of each stage is one month. The costs for NUT are a reasonable amount to buy healthy food for one participant per month in the Netherlands. The costs for PHY in the first set cover a subscription to the local gym for one month, those in the second set also include personal training by a fitness coach. Furthermore, the budget is B=100,000 . For the response rates and the weights, we consider the same sets of values as in Section ‘Locally optimal design under a fixed total sample size’.

For $\left(C_{N},C_{P})=(300,50\right)$ , the optimal proportion $p_{1}^{*}$ is somewhat above 0.5, which implies that in the first stage more subjects are randomized to the least expensive treatment PHY than to the more expensive treatment NUT. The optimal proportion $p_{1}^{*}$ hardly depends on the chosen weights, but it slightly increases with increasing response rates. Higher response rates imply more subjects receive the same treatment in stage 2 as they did in stage 1. It is therefore advantageous to already randomize more subjects to the least expensive treatment PHY in stage 1, so that more subjects receive this treatment in stage 2 as well. For $\left(C_{N},C_{P})=(300,300\right)$ , both first-stage treatments are equally expensive and the optimal proportion $p_{1}^{*}$ is (about) 0.5. It hardly depends on the chosen weights and the response rates.

The optimal proportions $p_{2}^{*}$ and $p_{3}^{*}$ hardly depend on the response rates but they do depend on the chosen weights. For the first set of weights, $\left(\lambda_{13},\lambda_{14},\lambda_{23},\lambda_{24})=(0.25,\;0.25,\;0.25,\;0.25\right)$ , somewhat more subjects are randomized to the single second-stage treatments NUT or PHY than to the combined second-stage treatment PHY + NUT. This is obvious since single second-stage treatments are less expensive than combined treatments. For the second set of weights, $\left(\lambda_{13},\lambda_{14},\lambda_{23},\lambda_{24})=(0.70,\;0.10,\;0.10,\;0.10\right)$ , even more subjects are randomized to single second-stage treatments than for the first set of weights. This is also obvious because the second set of weights puts more emphasis on the comparison of those ATSs with single second-stage treatments. For the third set of weights, $\left(\lambda_{13},\lambda_{14},\lambda_{23},\lambda_{24})=(0.10,\;0.10,\;0.10,\;0.70\right)$ , more subjects are randomized to combined second-stage treatments than to single second-stage treatments, which is also obvious because this set of weights puts more emphasis on the comparison of ATSs with combined second-stage treatments.

The optimal total sample size $N^{*}$ depends on the combination of costs $\left(C_{N},C_{P}\right)$ . As is obvious, fewer subjects can be included for $C_{P}=300$ than for $C_{P}=50$ . Furthermore, $N^{*}$ depends on the weights: most subjects can be included for the second set of weights and fewest for the third set of weights. For the second set of weights, more subjects are randomized to the least expensive single second-stage treatments, hence a larger total number of subjects can be included. Finally, more subjects can be included when the response rates increase. Subjects who respond to treatment are not re-randomized, hence they receive a single treatment in the second-stage. Single treatments are less expensive than combined treatments, hence more subjects can be included.

The RE of the balanced design slightly depends on the response rates. It is also related to the weights. The RE is highest for the first set of weights, since the optimal proportions are nearest to those of the balanced design. Slightly lower relative efficiencies are found for the third set of weights, but these relative efficiencies are still above 0.9. The lowest relative efficiencies are observed for the second set of weights as the optimal proportions deviate most from those of the balanced design. The lowest RE is RE=0.85 , which implies that the balanced design requires 100%[1−(1/0.85)]=17% more subjects than the optimal design.

### Robust optimal design

The optimal designs that were presented in subsections ‘Locally optimal design under a fixed total sample size’ and ‘Locally optimal design under a fixed budget’ are locally optimal since they depend on the response rates $\gamma_{1}$ and $\gamma_{2}$ . Such response rates are often unknown in the design phase of a SMART and an educated a priori guess must be given. There is, however, no guarantee such a guess is correct, and an incorrect guess may result in a suboptimal design. This problem may be overcome by using robust optimal design methodology; here we use the maximin optimal design methodology as described in section ‘Robust optimal design’.

Tables 5 and 6 in the online supplement show maximin optimal designs using the same sets of weights and combinations of costs as in Tables 3 and 4. The ranges used for the response rates are $\left[\gamma_{1}-0.05,\gamma_{1}+0.05\right]$ and $\left[\gamma_{2}-0.05,\gamma_{2}+0.05\right]$ , where $\gamma_{1}$ and $\gamma_{2}$ are the values in Tables 3 and 4.

**Table 4.** Locally optimal design: optimal proportions for first-stage $(p_{1}^{*})\;$ and second-stage $(p_{2}^{*},\;p_{3}^{*})\;$ treatments for three different sets of weights $\left(\lambda_{13},\lambda_{23},\lambda_{14},\lambda_{24}\right)$ for the multiple-objective optimal design and for three different sets of response rates $\left(\gamma_{1},\gamma_{2}\right)$ . The relative efficiency (RE) of the balanced design is also provided. The optimal proportions are derived under a fixed budget with C=100,000 and for two different sets of costs $\left(C_{P},C_{N}\right)$ .

		$(\lambda_{13},\lambda_{23},\lambda_{14},\lambda_{24})=$ (0.25,0.25,0.25,0.25)					$(\lambda_{13},\lambda_{23},\lambda_{14},\lambda_{24})=$ (0.70,0.10,0.10,0.10)					$(\lambda_{13},\lambda_{23},\lambda_{14},\lambda_{24})=$ (0.10,0.10,0.10,0.70)				
$\gamma_{1}$	$\gamma_{2}$	$p_{1}^{*}$	$p_{2}^{*}$	$p_{3}^{*}$	$N^{*}$	RE	$p_{1}^{*}$	$p_{2}^{*}$	$p_{3}^{*}$	$N^{*}$	RE	$p_{1}^{*}$	$p_{2}^{*}$	$p_{3}^{*}$	$N^{*}$	RE
$\left(C_{N},C_{P})=(300,50\right)$																
0.15	0.25	0.56	0.52	0.56	243	0.98	0.55	0.68	0.72	255	0.85	0.56	0.35	0.39	232	0.93
0.25	0.40	0.58	0.52	0.56	250	0.97	0.57	0.68	0.72	259	0.86	0.58	0.35	0.39	241	0.93
0.40	0.55	0.60	0.52	0.55	265	0.96	0.60	0.68	0.71	272	0.86	0.60	0.35	0.38	257	0.92
$\left(C_{N},C_{P})=(300,300\right)$																
0.15	0.25	0.50	0.55	0.55	141	0.99	0.50	0.71	0.71	149	0.85	0.50	0.38	0.38	133	0.96
0.25	0.40	0.51	0.55	0.54	144	0.99	0.51	0.71	0.71	152	0.87	0.51	0.38	0.38	138	0.96
0.40	0.55	0.51	0.54	0.54	149	1	0.51	0.70	0.70	155	0.89	0.51	0.38	0.37	143	0.96

A comparison of Table 3 and Table 5 of Supplemental material, and Table 4 and Table 6 of Supplemental material shows the locally optimal designs and maximin optimal designs are (almost) identical for the chosen sets of weights, response rates and costs. As a result, the minimal RE of the balanced design as given in Tables 5 and 6 of Supplemental material is almost equal to that of the RE of the balanced design in Tables 3 and 4. This result is not surprising since in Sections ‘Locally optimal design under a fixed total sample size’ and ‘Locally optimal design under a fixed budget’, it was shown that the optimal design hardly depends on the response rates. Of course, this finding does not necessarily hold for all combinations of responses rates, weights and costs. The user is therefore encouraged to apply maximin optimal design methodology in the case the response rates are likely to be misspecified.

## Discussion

Considering our example of a prototypical SMART design, we derived the optimal design $\xi^{*}=(\;p_{1}^{*},\;p_{2}^{*},\;p_{3}^{*})$ both under a fixed sample size and budget constraint. Under a fixed sample size, we found that the optimal probability in the first-stage $p_{1}^{*}$ is mostly influenced by the weights chosen for the multiple-objective optimal design, while it is only slightly influenced by the response rates. On the other hand, second-stage optimal probabilities are only influenced by the choice of the weights. When considering the second set of weights $\left(\lambda_{13},\;\lambda_{14},\;\lambda_{23},\lambda_{24})=(0.70,\;0.10,\;0.10,\;0.10\right)$ or the third set, $\left(\lambda_{13},\;\lambda_{14},\;\lambda_{23},\lambda_{24})=(0.10,\;0.10,\;0.10,\;0.70\right)$ , which, respectively, put more emphasis on the use of single and combined treatments, the optimal design $\xi^{*}\;$ performs better than the balanced design $\xi_{b}=(0.50,0.50,\;0.50)$ , although the latter still achieves a RE above 0.90. When equal weights are used, $\xi^{*}$ and $\xi_{b}$ perform almost identically in terms of RE.

Under a fixed budget, the optimal proportions are influenced also by the cost of treatments, besides the aforementioned weights and response rates. When including cost of treatments into account, the performance in terms of RE of the optimal design $\xi^{*}$ , with respect to $\xi_{b}$ , improves. The reason might be that unequal allocation of patients to intervention options seems to work better under a fixed budget than under a fixed sample size, as was also previously stated in the literature.^2,3^

It is especially advised to use the optimal design rather than the balanced design when the second set of weights, $\left(\lambda_{13},\;\lambda_{14},\;\lambda_{23},\lambda_{24})=(0.70,\;0.10,\;0.10,\;0.10\right)$ , is used. For this set, $\xi_{b}$ may have a RE as low as 0.86. When using equal weights for the multiple-objective optimal design, $\xi_{b}$ achieves a RE with respect to $\xi^{*}$ above 0.95. When using the third set of weights, $\left(\lambda_{13},\;\lambda_{14},\;\lambda_{23},\lambda_{24})=(0.10,\;0.10,\;0.10,\;0.70\right)$ , $\xi_{b}$ achieves a RE above 0.90.

It should be mentioned that the optimal designs are locally optimal, as they depend on the two unknown response rates $\gamma_{1}$ and $\gamma_{2}$ . One way to address this issue is using maximin optimal design methodology. In our example, the maximin optimal designs are quite similar to the locally optimal designs. In other words, the locally optimal designs are rather robust with respect to mild misspecification of the response rates. However, this finding does not always hold and it is advocated to derive a maximin optimal design if there is uncertainty about the a priori guesses of the response rates.

We derived our optimal design under the assumption that outcomes of subjects in ATSs that start with different first-stage treatments are independent of each other, resulting in a zero correlation between weighted mean outcomes of ATSs starting with different first-stage treatments. There are situations in which this assumption may be violated. Consider for instance the situation in our weight loss example where just a limited number of personal trainers is available. It may then occur, a personal trainer trains subjects from ATSs starting with different first-stage treatments. In such a case, the outcomes of subjects who have been trained by the same personal trainer become dependent because of the trainer's skills, enthusiasm, experience, etc. In such a case, the assumption of independence is violated and hence our optimal design is not applicable. Such a problem can be easily solved by letting each personal trainer only train subjects from ATSs that start with the same first-stage treatment.

One limitation of this study is that it does not take clustered data structures into account, while such data may also occur in SMARTs.^54,55^ Clustered data occur, among others, in cluster-randomized trials and multicentre trials. In such studies not only the total number of subjects in each treatment sequence needs to be determined, but also the number of clusters and cluster size.^56^ The optimal design will depend on the intraclass correlation coefficient, which measures the degree of dependence of outcomes within the same cluster.

Another limitation of this study is that formulae and methodology only apply to the prototypical SMART designs in Figures 1 and 2. Based on the number of treatments, stages and randomizations, different SMART designs can be developed, of which many examples exist in the literature^57,58^ and online.^59^ It would be necessary to study optimal designs for such other types of SMART designs.

To our knowledge, this is the first paper that studies optimal allocation to treatments in SMARTs. Our Shiny App allows researchers in the fields of biomedical, health and social sciences to derive the optimal design for their SMART and to calculate the efficiency of a balanced design. We hope that this paper will further contribute to the development and implementation of SMARTs.

## Supplemental Material

sj-docx-1-smm-10.1177_09622802211037066 - Supplemental material for Optimal allocation to treatments in a sequential multiple assignment randomized trial Click here for additional data file. Supplemental material, sj-docx-1-smm-10.1177_09622802211037066 for Optimal allocation to treatments in a sequential multiple assignment randomized trial by Andrea Morciano and Mirjam Moerbeek in Statistical Methods in Medical Research
