# Supplementary material for: Optimal allocation to treatments in a sequential multiple assignment randomized trial
Source: Stat Methods Med Res. 2021 Sep 23;30(11):2471–84. doi: 10.1177/09622802211037066 (PMC8649474; doi:10.1177/09622802211037066)
Supplement: sj-docx-1-smm-10.1177_09622802211037066 - Supplemental material for Optimal allocation to treatments in a sequential multiple assignment randomized trial [file sj-docx-1-smm-10.1177_09622802211037066.docx]

Optimal allocation to treatments

in a Sequential Multiple Assignment Randomized Trial (SMART)

# Online Supplement

# Derivation of the optimal design under a fixed sample size

Under a fixed budget, we search for the optimal design of the multiple-objective optimal design. As is shown in our paper, its formula is given by:

$$\begin{aligned} \Phi= \Phi_{13}\lambda_{13}+\Phi_{14}\lambda_{14}+ \Phi_{23}\lambda_{23}+\Phi_{24}\lambda_{24} , \#\left( 1 \right) \end{aligned}$$

with $\lambda_{ii^{'}}$ being the weight assigned to the respective objective $\Phi_{ii^{'}}$. By plugging in the respective objectives $\Phi_{ii^{'}}$, we obtain:

$$\Phi=\left[ \sigma^{2}\frac{\gamma_{1}p_{2}+\left( 1-\gamma_{1} \right)}{Np_{1}p_{2}}+\sigma^{2}\frac{\gamma_{2}p_{3}+\left( 1-\gamma_{2} \right)}{N\left( 1-p_{1} \right)p_{3}} \right]\lambda_{13}+\left[ \sigma^{2}\frac{\gamma_{1}p_{2}+\left( 1-\gamma_{1} \right)}{Np_{1}p_{2}}+\sigma^{2}\frac{\gamma_{2}\left( 1-p_{3} \right)+\left( 1-\gamma_{2} \right)}{N\left( 1-p_{1} \right)\left( 1-p_{3} \right)} \right]\lambda_{14}+$$

$\left[ \sigma^{2}\frac{\gamma_{1}\left( 1-p_{2} \right)+(1-\gamma_{1})}{Np_{1}(1-p_{2})}+\sigma^{2}\frac{\gamma_{2}p_{3}+(1-\gamma_{2})}{N(1-p_{1})p_{3}} \right]\lambda_{23}+\left[ \sigma^{2}\frac{\gamma_{1}\left( 1-p_{2} \right)+(1-\gamma_{1})}{Np_{1}(1-p_{2})}+\sigma^{2}\frac{\gamma_{2}\left( 1-p_{3} \right)+(1-\gamma_{2})}{N(1-p_{1})(1-p_{3})} \right]\lambda_{24}$.

In this setting, an analytical solution to find the optimal design was found by taking the gradient of $\Phi$ with respect to $p_{1}$, $p_{2}$, $p_{3}$. The gradient of $\Phi$ is given by:

$$\nabla\Phi=\frac{\partial\Phi}{\partial p_{1}}+\frac{\partial\Phi}{\partial p_{2}}+\frac{\partial\Phi}{\partial p_{3}},$$

Where

$$\frac{\partial\Phi}{\partial p_{1}}=\sigma^{2}\frac{\gamma_{2}p_{3}+\left( 1-\gamma_{2} \right)}{N\left( 1-p_{1} \right)^{2}p_{3}}\left( \lambda_{13}+\lambda_{23} \right)+\sigma^{2}\frac{\gamma_{2}{(1-p}_{3})+\left( 1-\gamma_{2} \right)}{N\left( 1-p_{1} \right)^{2}\left( 1-p_{3} \right)}\left( \lambda_{14}+\lambda_{24} \right)-\sigma^{2}\frac{\gamma_{1}p_{2}+\left( 1-\gamma_{1} \right)}{N{p_{1}}^{2} p_{2}}\left( \lambda_{13}+\lambda_{14} \right)$$

$-\sigma^{2}\frac{\gamma_{1}(1-p_{2})+\left( 1-\gamma_{1} \right)}{N{p_{1}}^{2} {(1-p}_{2})}\left( \lambda_{23}+\lambda_{24} \right)$,

$\frac{\partial\Phi}{\partial p_{2}}=\frac{\sigma^{2}\left( 1-\gamma_{1} \right)}{Np_{1}\left( 1-p_{2} \right)^{2}}\left( \lambda_{23}+\lambda_{24} \right)-\frac{\sigma^{2}\left( 1-\gamma_{1} \right)}{Np_{1}{p_{2}}^{2}}\left( \lambda_{13}+\lambda_{14} \right)$,

$\frac{\partial\Phi}{\partial p_{3}}=\frac{\sigma^{2}\left( 1-\gamma_{2} \right)}{N{(1-p}_{1})\left( 1-p_{3} \right)^{2}}\left( \lambda_{14}+\lambda_{24} \right)-\frac{\sigma^{2}\left( 1-\gamma_{2} \right)}{N(1-p_{1}){p_{3}}^{2}}\left( \lambda_{13}+\lambda_{23} \right)$,

We therefore set these first derivatives equal to 0, such that $\frac{\partial\Phi}{\partial p_{1}}=0$, $\frac{\partial\Phi}{\partial p_{2}}=0$ and $\frac{\partial\Phi}{\partial p_{3}}=0$, in order to find the minimum of $\Phi$ with respect to $p_{1}, p_{2}, p_{3}$. From this, it follows:

$$\frac{\partial\Phi}{\partial p_{1}}=0 \Rightarrow\frac{\gamma_{2}p_{3}+\left( 1-\gamma_{2} \right)}{\left( 1-p_{1} \right)^{2}p_{3}}\left( \lambda_{13}+\lambda_{23} \right)+\frac{\gamma_{2}{(1-p}_{3})+\left( 1-\gamma_{2} \right)}{\left( 1-p_{1} \right)^{2}\left( 1-p_{3} \right)}\left( \lambda_{14}+\lambda_{24} \right)$$

$$-\frac{\gamma_{1}p_{2}+\left( 1-\gamma_{1} \right)}{{p_{1}}^{2} p_{2}}\left( \lambda_{13}+\lambda_{14} \right)-\frac{\gamma_{1}{(1-p}_{2})+\left( 1-\gamma_{1} \right)}{{p_{1}}^{2} (1-p_{2})}\left( \lambda_{23}+\lambda_{24} \right)=0$$

$$\frac{\partial\Phi}{\partial p_{2}}=0 \Rightarrow\frac{\left( \lambda_{23}+\lambda_{24} \right)}{\left( 1-p_{2} \right)^{2}}-\frac{\left( \lambda_{13}+\lambda_{14} \right)}{{p_{2}}^{2}}=0$$

$$\frac{\partial\Phi}{\partial p_{3}}=0 \Rightarrow\frac{\left( \lambda_{14}+\lambda_{24} \right)}{\left( 1-p_{3} \right)^{2}}-\frac{\left( \lambda_{13}+\lambda_{23} \right)}{{p_{3}}^{2}}=0$$

The solutions to these equations yield the locally optimal design. These are given by:

$$p_{1}^{*}=\frac{\sqrt{\varphi}}{\sqrt{\psi}+\sqrt{\varphi}}$$

Where

$$\psi= \left[ \left( \gamma_{2}p_{3}^{*}+\left( 1-\gamma_{2} \right) \right)\left( \lambda_{13}+\lambda_{23} \right)\left( 1-p_{3}^{*} \right)+\left( \gamma_{2}\left( 1-p_{3}^{*} \right)+\left( 1-\gamma_{2} \right) \right)\left( \lambda_{14}+\lambda_{24} \right)p_{3}^{*} \right]\times p_{2}^{*}\left( 1-p_{2}^{*} \right)$$

$$\varphi= \left[ \left( \gamma_{1}p_{2}^{*}+\left( 1-\gamma_{1} \right) \right)\left( \lambda_{13}+\lambda_{14} \right)\left( 1-p_{2}^{*} \right)+\left( \gamma_{1}\left( 1-p_{2}^{*} \right)+\left( 1-\gamma_{1} \right) \right)\left( \lambda_{23}+\lambda_{24} \right)p_{2}^{*} \right]\times p_{3}^{*}\left( 1-p_{3}^{*} \right)$$

$$p_{2}^{*}=\frac{\sqrt{\left( \lambda_{13}+\lambda_{14} \right)}}{\sqrt{\left( \lambda_{23}+\lambda_{24} \right)}+\sqrt{\left( \lambda_{13}+\lambda_{14} \right)}}$$

$$p_{3}^{*}=\frac{\sqrt{\left( \lambda_{13}+\lambda_{23} \right)}}{\sqrt{\left( \lambda_{13}+\lambda_{23} \right)}+\sqrt{\left( \lambda_{14}+\lambda_{24} \right)}}$$

# Domain search algorithm

As seen in our paper, under a fixed budget, an analytical solution for the optimal design for the multiple-objective optimal design cannot be found. However, a numerical solution through a domain search algorithm can be found. As $p_{1}$, $p_{2}$ and $p_{3}$ are probabilities, their domain is defined over [0, 1]. The algorithm involves four steps:

1. We use as the initial interval the domain of $p_{1}$, $p_{2}$ and $p_{3}$, which we consider to be discrete instead of being continuous. Over the newly discretized interval, a step size of 0.02 is chosen in evaluating combinations. Also, a counter variable $i$ is created and initialized to 1.
2. The value of the function in (1) is evaluated for all possible combinations of $p_{1}$, $p_{2}$ and $p_{3}$ in the interval, given the weights, response rates, cost of treatments and budget.
3. The combination of $p_{1}$, $p_{2}$ and $p_{3}$ that provides the minimum value for (9) is selected as the temporary optimal design, with $p_{1}^{'}, p_{2}^{'}, p_{3}^{'}$ denoting the temporary optimal probabilities. Also, $i=i+1$.
4. A new interval for $p_{1}$, $p_{2}$ and $p_{3}$ is obtained, such that $p_{1}\in[p_{1}^{'}-k, p_{1}^{'}+k]$, $p_{2}\in[p_{2}^{'}-k, p_{2}^{'}+k]$, $p_{3}\in[p_{3}^{'}-k, p_{3}^{'}+k]$ with $k$ depending on the value of $i$. Specifically, if $i=2$, $k=0.18$, while if $i\neq2, k=3.5*{10}^{-(i-1)}$. Also, the step size used depends on $i$. When $i=2$, we use as step size $0.03$, while when $i\neq2$, we use as step size $2*{10}^{-i}$.

Steps 2 – 4 are repeated iteratively, until a convergence criterion is met. Here convergence is met when the derived temporary optimal values $p_{1}^{'}, p_{2}^{'}, p_{3}^{'}$ are the same in two subsequent iterations with a precision of $2.013*{10}^{-5}$.

# Calculation of total sample size from budget, costs and optimal proportions

Equation (15) in the main document shows the relation between budget, costs and sample sizes:

$$C= c_{A}N_{A}+c_{B}N_{B}+\ldots+c_{H}N_{H}.$$

Using equations (16)-(18), the sample sizes $N_{A}$, …, $N_{H}$ are replaced by their expected values:

$$C= c_{A}p_{1}N+c_{B}\left( 1-p_{1} \right)N+c_{C}\gamma_{1}p_{1}N+c_{D}\gamma_{1}p_{1}p_{2}N+c_{E}\gamma_{1}p_{1}\left( 1-p_{2} \right)N+$$

$c_{F}\gamma_{2}\left( 1-p_{1} \right)N+c_{G}\gamma_{2}\left( 1-p_{1} \right)p_{3}N+c_{H}\gamma_{2}(1-p_{1})\left( 1-p_{3} \right)N$.

From this equation we can calculate the total sample size:

$N=\frac{C}{c_{A}p_{1}+c_{B}\left( 1-p_{1} \right)+c_{C}\gamma_{1}p_{1}+c_{D}\gamma_{1}p_{1}p_{2}+c_{E}\gamma_{1}p_{1}\left( 1-p_{2} \right)+c_{F}\gamma_{2}(1-p_{1})+c_{G}\gamma_{2}(1-p_{1})p_{3}+c_{H}\gamma_{2}(1-p_{1})\left( 1-p_{3} \right)}$.

# Maximin Optimal Design

**Table 5.** Maximin optimal design: optimal proportions for first-stage ($p_{1}^{*})$and second-stage ($p_{2}^{*}, p_{3}^{*})$treatments for three different sets of weights $(\lambda_{13},\lambda_{23},\lambda_{14},\lambda_{24})$ for the multiple-objective optimal design, and for three different sets of response rates $(\gamma_{1},\gamma_{2})$. The relative efficiency (RE) of the balanced design is also provided. The optimal proportions are derived under a fixed total sample size.

|  |  |  | $\left( \lambda_{13},\lambda_{23},\lambda_{14},\lambda_{24} \right)=$  $(0.25, 0.25, 0.25, 0.25)$ | | | |  | $\left( \lambda_{13},\lambda_{23},\lambda_{14},\lambda_{24} \right)=$  $(0.70, 0.10, 0.10, 0.10)$ | | | |  | $\left( \lambda_{13},\lambda_{23},\lambda_{14},\lambda_{24} \right)=$  $(0.10, 0.10, 0.10, 0.70)$ | | | |
| --- | --- | --- | --- | --- | --- | --- | --- | --- | --- | --- | --- | --- | --- | --- | --- | --- |
| $\gamma_{1}$ | $\gamma_{2}$ |  | $p_{1}^{*}$ | $p_{2}^{*}$ | $p_{3}^{*}$ | minRE |  | $p_{1}^{*}$ | $p_{2}^{*}$ | $p_{3}^{*}$ | minRE |  | $p_{1}^{*}$ | $p_{2}^{*}$ | $p_{3}^{*}$ | minRE |
| [0.10, 0.20] | [0.20, 0.30] |  | 0.50 | 0.50 | 0.50 | 1 |  | 0.50 | 0.65 | 0.65 | 0.91 |  | 0.50 | 0.35 | 0.35 | 0.91 |
| [0.20, 0.30] | [0.35, 0.45] |  | 0.50 | 0.50 | 0.50 | 1 |  | 0.51 | 0.67 | 0.67 | 0.92 |  | 0.50 | 0.35 | 0.35 | 0.92 |
| [0.35, 0.45] | [0.50, 0.60] |  | 0.50 | 0.50 | 0.50 | 1 |  | 0.50 | 0.65 | 0.65 | 0.93 |  | 0.50 | 0.35 | 0.35 | 0.92 |

**Table 6.** Maximin optimal design: optimal proportions for first-stage ($p_{1}^{*})$and second-stage ($p_{2}^{*}, p_{3}^{*})$treatments for three different sets of weights $(\lambda_{13},\lambda_{23},\lambda_{14},\lambda_{24})$ for the multiple-objective optimal design, and for three different sets of response rates $(\gamma_{1},\gamma_{2})$. The relative efficiency (RE) of the balanced design is also provided. The optimal proportions are derived under a fixed budget with $C=100,000$ and for two different sets of costs $(C_{P},C_{N})$.

|  |  |  | $\left( \lambda_{13},\lambda_{23},\lambda_{14},\lambda_{24} \right)=$  $(0.25, 0.25, 0.25, 0.25)$ | | | | |  | $\left( \lambda_{13},\lambda_{23},\lambda_{14},\lambda_{24} \right)=$  $(0.70, 0.10, 0.10, 0.10)$ | | | | |  | $\left( \lambda_{13},\lambda_{23},\lambda_{14},\lambda_{24} \right)=$  $(0.10, 0.10, 0.10, 0.70)$ | | | | |
| --- | --- | --- | --- | --- | --- | --- | --- | --- | --- | --- | --- | --- | --- | --- | --- | --- | --- | --- | --- |
| $\gamma_{1}$ | $\gamma_{2}$ |  | $p_{1}^{*}$ | $p_{2}^{*}$ | $p_{3}^{*}$ | $N^{*}$ | minRE |  | $p_{1}^{*}$ | $p_{2}^{*}$ | $p_{3}^{*}$ | $N^{*}$ | minRE |  | $p_{1}^{*}$ | $p_{2}^{*}$ | $p_{3}^{*}$ | $N^{*}$ | minRE |
| ${(C}_{N},C_{P})=\left( 300,50 \right)$ | | | | | | | | | | | | | | | | | | | |
| [0.10, 0.20] | [0.20, 0.30] |  | 0.55 | 0.50 | 0.55 | 238 | 0.98 |  | 0.55 | 0.69 | 0.73 | 257 | 0.84 |  | 0.55 | 0.35 | 0.40 | 228 | 0.93 |
| [0.20, 0.30] | [0.35, 0.45] |  | 0.60 | 0.50 | 0.55 | 249 | 0.97 |  | 0.57 | 0.68 | 0.71 | 262 | 0.86 |  | 0.60 | 0.35 | 0.40 | 240 | 0.93 |
| [0.35, 0.45] | [0.50, 0.60] |  | 0.60 | 0.50 | 0.55 | 260 | 0.95 |  | 0.60 | 0.70 | 0.70 | 269 | 0.86 |  | 0.60 | 0.35 | 0.40 | 252 | 0.92 |
| $(C_{N},C_{P})=\left( 300,300 \right)$ | | | | | | | | | | | | | | | | | | | |
| [0.10, 0.20] | [0.20, 0.30] |  | 0.50 | 0.55 | 0.55 | 140 | 0.99 |  | 0.50 | 0.70 | 0.70 | 148 | 0.85 |  | 0.50 | 0.40 | 0.40 | 133 | 0.96 |
| [0.20, 0.30] | [0.35, 0.45] |  | 0.50 | 0.55 | 0.55 | 143 | 0.99 |  | 0.50 | 0.70 | 0.70 | 150 | 0.86 |  | 0.50 | 0.40 | 0.40 | 137 | 0.96 |
| [0.35, 0.45] | [0.50, 0.60] |  | 0.50 | 0.55 | 0.55 | 148 | 1 |  | 0.50 | 0.70 | 0.70 | 153 | 0.88 |  | 0.50 | 0.35 | 0.35 | 140 | 0.96 |
